# Ovariotomy—Removal of a Multilocular Ovarian Tumor, Weighing Nineteen and a Half Pounds, with Fatal Results

**Published:** 1866-09

**Authors:** J. F. Miner


ART. II.—*Ovariotomy—Removal of a Multilocular Ovarian Tumor, weighing nineteen and a half pounds, with fatal results.* By J. F. Miner, M. D.

In estimating the propriety of an operation, as well as in selecting the most safe and desirable manner of making it, reports of unfavorable cases are of no less value than successful ones, and in some respects there are more cogent reasons for publishing unfavorable than successful operations.

Ovariotomy has not yet attained to such an established acknowledgment that its propriety is not questioned; and the only way its merits can be fully tested is by impartial history. It would seem that its achievements were already sufficient to convince the most obstinate of its value, but this is not wholly true until surgeons unanimously come to regard it as desirable under favorable circumstances, and recommend it unconditionally as a procedure offering reasonable chances of success—recommend it with hope und confidence. That it is unattended by great risk and danger, even in the best cases, is not claimed, but that its general results fully establish its benefits, and that it offers the only grounds of hope. The exact ratio of mortality is not yet established, cannot be determined at present; and will doubtless always be found to vary with the widely varying circumstances attending the disease, and the manner and time of its removal.

The following case is interesting in all respects, and is reported in detail, with the view that one faithfully reported case terminating fatally, is more instructive and of greater value in determining unsettled points than one which has recovered:

July 10, 1866, Miss Sexsmith, aged 32, presented herself for operation, and gave the following history: Was in good health until about two years since, when the right side commenced to enlarge, became tender, and at times painful, and these symptoms have gradually increased in severity and importance until the present time. Menstruation has been regular and natural, and the general health pretty good, with the exception of the pain and inconvenience connected with the growth of the abdomen.

Upon examination the abdomen was found distended to its full capacity, the tumor somewhat irregular, laying more to the right side than otherwise, hard, unyielding, and apparently attached so as to admit of very little if any movement of the abdominal walls upon the growth. She had never been tapped, and the diagnosis appeared so satisfactory without exploratory measures of this sort, that this means of determining its character was neglected.—Future developments proved how little value and how liable to deceive such a measure would have proved, the thick contents of the sacs not readily passing through a canula of one-half inch diameter.

The patient fully anæsthetized, the following operation was made in presence of and assisted by a large number of professional friends: Incision was made about three inches in extent down upon the tumor; very little fluid was contained in abdominal cavity outside the cysts. The hand was carefully introduced and the tumor found attached throughout its whole anterior surface to the peritoneum, by apparently recent attachments which were readily and easily broken. Trocar and canula was now introduced with the view to lessen the tumor and allow its extirpation through as small an opening as possible, in the abdominal walls. A large ovarian trocar was introduced and through the canula, after considerable time, oozed the thick, yellow, soap-like fluid, which comprised the contents of the two principal sacs opened. By this means the tumor was reduced to a mass weighing about five pounds, and elevated from its bed through an incision not over three inches in extent; care was taken that neither blood or the contents of the cysts should flow into the abdominal cavity. The pedicle was found small, and was ligated by a single silk cord and the extremities were then passed through the vaginal septum, as proposed in a report of a successful case, published in the July number, where the vessels of the cord were tied separately and the ligatures cut closely. It may here be remarked, that this procedure though adopted in a case which did not recover, still appeared to work favorably, and nothing can be urged against it upon the ground of failure, as it could have had no unfavorable influence.

The pedicle was returned after its ligation, and division with the eccraseur into the abdominal cavity, and the wound brought together with silver sutures introduced deeply, so as to inclose the edges of the peritoneum; water dressings, compress and bandage applied, and one-half grain morphine given subcutaneously.

The patient soon roused from the influence of the ether and appeared as well as if no operation had been made.

July 11th.—This morning appears quite cheerful, has had a very good night; vomited the evening after the operation, but not since. Pulse 84, and normal, good relish for food. Urine drawn with catheter. *Evening;* pulse 88, otherwise no change.

July 12th.—Pulse 96 per minute; no pain; some thirst; urinates naturally. Directed to take one-half teaspoonful of tinc. opium every six hours if in pain.

July 13th.—Pulse 96 per minute; urinates freely; has some relish for food and less thirst; some pain in the lower abdomen and tympanitis. Continued opium, food, and general support.

July 14th.—Skin cool; vomits occasionally, and complains of prostration. *Evening;* pulse 120, bowels tender and tympanitic; thirst, but no chill, no nausea since morning; bowels moved freely, and urine passed naturally.

July 15th.—Pulse 108, slept well, no nausea, no chill, urinates naturally, desires some food, complains of pain and soreness in the abdomen; sufficient morphine administered to relieve all pain, requiring about one-half grain every four hours.

July 16th.—Has taken one-half grain morphine every four hours, but still suffered pain in the abdomen; pulse 96; bowels more distended and painful; otherwise unchanged.

July 17th.—Pulse 96 per minute, tongue clean, abdomen distended and tender; no nausea or vomiting.

July 18th.—Pulse 108 per minute and losing in force; abdomen fully distended, painful and tender; low delirium, with the complaint of seeing herself another; urinates naturally; complains of but little pain; continues one-half grain morphine every four hours and general support.

July 19th.—Pulse 108; tongue dry; slept last night for the most time; takes a little food, also morphine gr. ss every three hours, but still appears restless, and is evidently losing strength. *Evening*—Pulse have rapidly increased in frequency, and are now 136 per minute; constant thirst, great restlessness, appearing in every way distressed; abdomen tender, distended and tympanitic.

July 20th.—Appears more comfortable; slept very well, urinates naturally; takes some food and retains it; pulse 120.

July 21st.—Complains greatly of pain in the abdomen, and appears every way much worse.

July 22d.—Bowels move frequently as from attack of diarrhœa; vomits often, retaining nothing upon the stomach; depression very great; pulse weak, and 120 per minute.

July 23d.—Looks better; diarrhœa abated, though stools are still frequent; pulse 120, and more distinct and forcible; mind quite clear, and expresses hopes of recovery.

July 24th.—Exhausted; features sunken, skin cool and moist; pulse rapid, almost indistinct. Died at 2 o'clock P. M., fourteen days after the operation.

No *post mortem* examination could be obtained on account of removal, but slight exploration showed the incision completely healed; the bowels distended by gas, and no fluid in the abdominal cavity. There was no appearance of suppuration—no pus had discharged by the side of the ligature passed out through the vaginal septum.

There can be no doubt that the cause of death was diffused inflammation of the peritoneum. That the attachment to this membrane was instrumental in producing this result is probable, as the tumor over the whole anterior portion was closely connected to the peritoneum, and was separated at the time of operation.

It was remarkable that for the first few days no symptoms of disturbance should show themselves; the patient regarded herself as well, and I am since informed committed many imprudences, sitting up to comb her hair, and in other ways doing very unnecessary and imprudent things, for her own or others' benefit. I am, however, not aware of anything being done or left undone which could certainly influence her recovery, and regard the termination as dependent upon influences over which we could have no control.

